# Correlation between red blood cell transfusion volume and mortality in patients with massive blood transfusion: A large multicenter retrospective study

**DOI:** 10.3892/etm.2014.2068

**Published:** 2014-11-12

**Authors:** JIANG-CUN YANG, YANG SUN, CUI-XIANG XU, QIAN-LI DANG, LING LI, YONG-GANG XU, YAO-JUN SONG, HONG YAN

**Affiliations:** 1 Department of Transfusion Medicine, The Third Affiliated Hospital of Medical College of Xi’an Jiaotong University, Xi’an, Shaanxi 710061, P.R. China; 2 Shaanxi Provincial Center for Clinical Laboratory, The Third Affiliated Hospital of Medical College of Xi’an Jiaotong University, Xi’an, Shaanxi 710061, P.R. China; 3 Department of Dermatology, The Third Affiliated Hospital of Medical College of Xi’an Jiaotong University, Xi’an, Shaanxi 710061, P.R. China; 4 Department of Laboratory, The Third Affiliated Hospital of Medical College of Xi’an Jiaotong University, Xi’an, Shaanxi 710061, P.R. China; 5 Department of Urology, The Third Affiliated Hospital of Medical College of Xi’an Jiaotong University, Xi’an, Shaanxi 710061, P.R. China; 6 Department of Epidemiology and Health Statistics, Medical College of Xi’an Jiaotong University, Xi’an, Shaanxi 710061, P.R. China

**Keywords:** massive blood transfusion, red blood cell, mortality, retrospective analysis, multicenter

## Abstract

This study aimed to explore the correlation between red blood cell (RBC) transfusion volume and patient mortality in massive blood transfusion. A multicenter retrospective study was carried out on 1,601 surgical inpatients who received massive blood transfusion in 20 large comprehensive hospitals in China. According to RBC transfusion volume and duration, the patients were divided into groups as follows: 0–4, 5–9, 10–14, 15–19, 20–24, 25–29, 30–39 and ≥40 units within 24 or 72 h. Mortality in patients with different RBC transfusion volumes was analyzed. It was found that patient mortality increased with the increase in the volume of RBC transfusion when the total RBC transfusion volume was ≥10 units within 24 or 72 h. Survival analysis revealed significant differences in mortality according to the RBC transfusion volume (χ^2^=72.857, P<0.001). Logistic regression analysis revealed that RBC transfusion volume is an independent risk factor [odds ratio (OR) = 0.52; confidence interval (CI): 0.43–0.64; P<0.01] for the mortality of patients undergoing a massive blood transfusion. When RBCs were transfused at a volume of 5–9 units within 24 and 72 h, the mortality rate was the lowest, at 3.7 and 2.3% respectively. It is concluded that during massive blood transfusion in surgical inpatients, there is a correlation between RBC transfusion volume within 24 or 72 h and the mortality of the patients. Patient mortality increases with the increase in the volume of RBC transfusion. RBC transfusion volume, the length of stay at hospital and intensive care unit stay constitute the independent risk factors for patient mortality.

## Introduction

Massive blood transfusion is commonly defined as the administration of ≥10 units of packed red blood cells (pRBCs) to an individual patient (1,2) or the transfusion of more than one blood volume in 24 h (1,3–5). Alternative definitions include a ≥50% loss in blood volume within 3 h or a rate of loss of 150 ml blood/min in the severe traumatic and emergent situations (3). Massive blood transfusion is often provided to those who are injured during military operations, who have multiple injuries due to other causes, and who undergo complex surgery. A rational blood transfusion can improve the outcome of surgery, whereas unreasonable transfusion can increase mortality in patients.

Transfusion plays a key role in saving the lives of patients who have suffered massive blood loss. However, studies have found that mortality remains high for trauma patients who have received massive blood transfusion and suggest that there is a certain correlation between RBC transfusion volume and the mortality of patients (6–8). Stanworth *et al* (9) found that the mortality of patients who had received a pRBC transfusion was 9% for 0–5 units, 22% for 6–9 units and 42% for ≥10 units. Thus, it is necessary to maintain a balance between the advantages and disadvantages of RBC transfusion during massive blood transfusion.

Therefore, a multicenter retrospective study was conducted on cases of massive blood transfusion in 20 comprehensive hospitals from different regions of China to explore the correlation between RBC volume and the mortality of surgical inpatients with massive blood transfusion.

## Materials and methods

### Study protocol

This study was retrospective in nature. Data were collected from the medical records of surgical inpatients who received massive transfusion at 20 large-scale hospitals between January 2009 and December 2010. Between June 2010 and January 2011, 2,000 copies of the Massive Transfusion Survey Table (hereafter referred to as the Survey Table) were distributed to 20 Class III comprehensive hospitals in the northwest, southwest, central south, north and northeast regions of China. Members of the National Massive Transfusion Current Status Investigation Coordination Group (hereafter referred to as the Coordination Group) were responsible for collecting the data from these hospitals using the Survey Table. The data analysis was conducted at Shaanxi Provincial People’s Hospital, which is the Third Affiliated Hospital of the Medical College of Xi’an Jiaotong University (Xi’an, China). The present study was approved by the ethics committee of Xi’an Jiaotong University.

### Study population

Patients who received a transfusion of ≥10 units of pRBCs over a period of ≤24 h for trauma, cardiac surgery, obstetric conditions or other common surgeries (for example, orthopedic, thoracic, general, urinary, hepatobiliary and neurological surgery) were included in the study. By contrast, patients with coagulation disorders, hepatic failure due to medical causes, and coagulopathies were excluded from the analysis. Patients who received transfusions of <10 units for ≤24 h were assigned to the control group. Informed consent was obtained from the patients or the patients’ families prior to their inclusion in the current study.

### Survey table

The directors of the transfusion departments of the 20 participating hospitals discussed the topic, consulted experts and designed the Survey Table with reference to several international and domestic sources, in accordance with the principles of equality, voluntariness and mutual benefits. A meeting of the Coordination Group was then held, where 35 experts of clinical transfusion, surgery, anesthesia, gynecology and obstetrics, hematology and medical statistics discussed the study protocol and mode of data collection and also perfected and added supplements to the Survey Table. Suitable training was then offered to the investigating staff.

### Components of the survey table

The survey Table comprised four sections, as follows: i) Clinical and demographic characteristics of the patient, including name, gender, age, body weight, blood type, ethnicity, admission number, admission department, primary diagnosis, secondary diagnosis, pathologic diagnosis, nature of surgery and vital signs on admission. ii) Details regarding the perioperative complications, clinical condition within 24 h and after 24 h of the transfusion, and the total amount of blood transfused. iii) The results of the following blood tests performed before, within 24 h and after 24 h of transfusion: routine blood test, coagulation tests, liver function test, kidney function test, and arterial blood gas analysis. iv) Adverse events due to massive transfusion.

### Quality control

The Survey Table was first subjected to a small-scale preliminary test at Shaanxi Provincial People’s Hospital so that revisions could be made on the basis of the results and comments by experts to further improve the Table. One unit of pRBCs was derived from 200 ml whole blood and had a volume of 140–172 ml. One unit of fresh frozen plasma (FFP) was derived from 200 ml whole blood and had a volume of 100 ml. One bag of apheresis platelet was 10 units, and had a volume of 150–250 ml. One unit of platelet concentrate was derived from 200 ml whole blood and had a volume of 20–30 ml. The pRBCs were stored at 2–6°C. FFP was stored at ≤−18°C and thawed in a 37°C water bath, for ~10–15 min. Platelets were stored at 20–24°C in a platelet shaker.

### Data collected with devices and reagents

The main test devices and reagents used were as follows: Sysmex XE-2100 or XT-1800i hematology analyzer (Sysmex Corporation, Kobe, Japan); Coulter LH780 Coulter Hematology Analyzer (Beckman Coulter, Brea, CA, USA); Hitachi 7170A or 7180 Biochemical Analyzer (Hitachi, Tokyo, Japan); Roche Modular DP Automatic Biochemical Analyzer (Roche Diagnostics, Indianapolis, IN, USA); Olympus AU640 Biochemical Analyzer, (Olympus Corporation, Tokyo, Japan); Radiometer ABL-77 Blood Gas Analyzer (Radiometer, Copenhagen, Denmark); Roche Cobas- B123 Blood Gas Analyzer (Roche Diagnostics); and Sysmex CA1500/CA7000 Automatic Blood Coagulation Analyzer (Sysmex Corporation). All test reagents used were device-supporting reagents.

Data on the blood tests performed were collected from the laboratory records. These included: blood routine, coagulation tests, liver function test, kidney function and blood gas analysis. The data were collected for the blood tests performed prior to transfusion and at 16 different units during the 24-h transfusion (2U, 4U, 6U, 8U, 10U, 12U, 14U, 16U, 18U, 20U, 22U, 24U, 26U, 28U, 30U and 40U) and subjected to statistical analysis. The tests were conducted at the laboratory of each participating hospital, each of which undergoes internal quality control and an external quality assessment conducted by the Clinical Test Center of the Ministry of Health.

### Statistical analysis

Statistical analysis was conducted using SPSS software, version 18.0 (SPSS, Inc., Chicago, IL, USA). Epidata version 3.01 (Epidata Association, Odense, Denmark) was used for double data entry verification and database construction. The data on the demographic characteristics and clinical features were expressed as means with standard deviations or as absolute numbers. Categorical variables were analyzed by χ^2^ test, while continuous variables with normal distribution were analyzed by the Shapiro-Wilk test, analysis of variance, or the Kruskall-Wallis test, as appropriate. The Bonferroni method was applied for post-hoc tests to determine the significance of the differences between the group that received massive transfusion and the control group that did not. Linear regression was used to describe the relation between the number of units of pRBCs transfused and the platelet count. P<0.05 was considered to indicate a statistically significant result.

## Results

### Survey results

In total, 1,753 copies of the Survey Table were received from the 20 hospitals and the recovery rate was 87.65% (1,753/2,000). Of these, 1,601 copies were qualified tables without missing items and the qualification rate was 91.33% (1,601/1,753). The demographics and clinical data for the various RBC transfusion volume groups are shown in Table I. Among the 1,601 massive blood transfusion patients, 268 patients had undergone trauma (mortality, 34; survival, 234; mortality rate, 12.69%), 383 patients had undergone cardiac surgery (mortality, 53; survival, 330; mortality rate, 13.84%), 876 patients had undergone general surgery (mortality, 42; survival, 834; mortality rate, 4.79%) and 74 patients were obstetric patients (mortality, 3; survival, 71; mortality rate, 4.05%).

### Patient mortality

The mortality of the patients increased with the increase in the volume of RBC transfusion when the total RBC transfusion was >10 units, regardless of whether this was within 24 or 72 h. Within 24 h, as the volume of transfused RBCs increased from 10 to 40 units, the mortality rate rose from 6.0 to 38.9%. Within 72 h, as the volume of RBCs increased from 10 to 40 units, the mortality rate rose from 5.2% to 28.0%. When the volume of transfused RBCs was 5–9 units within 24 and 72 h, the mortality rate was the lowest, which was 3.7 and 2.3%, respectively. For transfusion with 0–4 units, the mortality rates were 7.3 and 9.7%, respectively (Table II).

**Figure 1:**
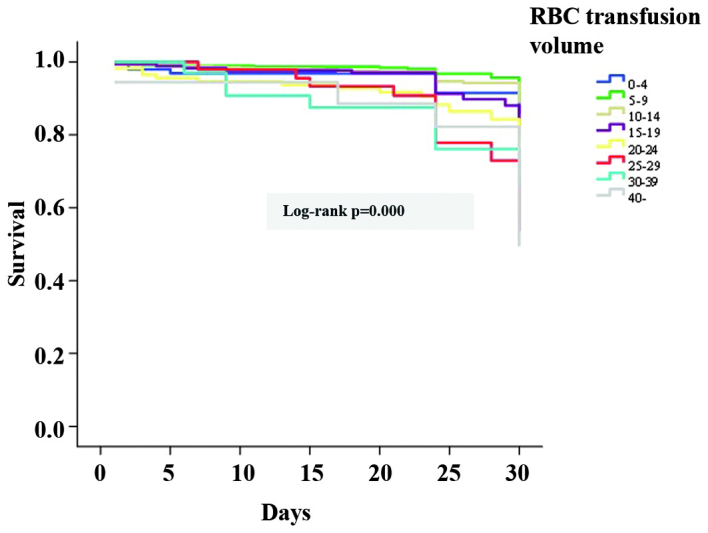
Kaplan-Meier survival chart of different red blood cell (RBC) transfusion volume groups.

Survival analysis showed that there were significant differences in mortality among the patients according to the RBC transfusion volume (χ^2^=72.857, P<0.001; Fig 1).

### Logistic regression analysis

Multivariate logistic regression analysis was performed with hospital mortality as the dependent variable. The following variables were considered as independent predictors: i) age, ii) gender, iii) surgery duration, iv) weight, v) length of stay in hospital, vi) intensive care unit (ICU) stay, vii) RBC volume (in 24 h) and viii) FFP volume (in 24 h). The results are presented as odds ratios (ORs) with 95% confidence intervals (95% CI; Table III). The factors that were identified to be significantly correlated with mortality were RBC volume (OR = 0.52, 95% CI: 0.43–0.64; P<0.001), length of stay in hospital (OR = 2.79; 95% CI: 1.31–5.92; P=0.01), and ICU stay (OR = 0.43; 95% CI; 0.21–0.88; P=0.02).

## Discussion

Transfusion plays an important role in saving the lives of patients in emergency and danger. Timely and sufficient blood transfusion is critical for the survival of patients who have suffered massive blood loss. Survival rates following massive transfusion have significantly increased in recent years. However, massive transfusion protocols have not always been associated with improved mortality (10). Long *et al* (11) examined the impact of postoperative hematocrit as an indicator of survival following massive transfusion in the trauma patient. They found that transfusion to hematocrits between 29.1 and 39% conveyed a survival benefit, whereas resuscitation to supraphysiologic hematocrits ≥39% conveyed no additional survival benefit. Sharpe *et al* (12) evaluated the effect of the number of RBC units transfused on the plasma:RBC and platelet:RBC ratios and their association with mortality in patients receiving massive transfusion. The authors found that patients receiving relatively higher quantities of RBCs were more likely to have a lower plasma:RBC ratio and were more likely to die.

The present study found that among 1,048 patients who received ≥10 units RBC transfusion volume within 24 h, the mortality rate was 10.31%, which is lower than the rate observed in related studies (9,13). This may be because the 20 medical institutions that participated in the present study are large general hospitals with better conditions, or because fewer trauma cases and a greater proportion of general surgery cases with good pre-operation preparation were included in this study, or due to the immediate application of fresh frozen plasma with a high percentage accompanying the RBC transfusion to correct coagulation at an initial stage; further research is required to clarify this. The present study also found that if the patients were classified by clinical department, the mortality of cardiac surgery and trauma patients receiving massive blood transfusion was 13.84 and 12.69% respectively, which is higher than the 4.79 and 4.05% mortality of general surgery and obstetrics patients, respectively.

According to previous studies, there is some correlation between RBC transfusion volume and the mortality of patients. The study conducted by Como *et al* (8) demonstrated that the mortality rate of 147 patients with massive blood transfusion was up to 39% and the mortality rate was 51% for patients receiving >50 units of blood products transfused within 24 h. Stanworth *et al* (9) indicated that the mortality rate was 9% when patients receiving 0–5 units pRBC transfusion, 22% for those receiving 6–9 units pRBCs and 42% for those receiving ≥10 units pBRCs. Surgenor *et al* (13) reported that RBC transfusion during or following cardiac surgery showed a certain correlation with the increase in the mortality of patients. The long-term risk of mortality was 16%, which was higher than that of patients undergoing transfusion with 1 or 2 units of pRBCs. The results of the present study for 1,601 surgical inpatients with transfusion were consistent with these previous studies. The present study also identified that a correlation existed between RBC transfusion volume and patient mortality. The multivariate logistic regression analysis results indicated that RBC transfusion volume (in 24 h), length of stay and ICU stay constitute independent risk factors for patient mortality.

However, certain limitations existed in this study. It was a large multicenter retrospective study, with a review of registry data in which a variable proportion of records may have been missing data. This is inevitable to a degree in analyses of multiple registries.

In summary, the present study highlights the correlation between RBC transfusion volume and patient mortality for surgical inpatients with massive blood transfusion. The mortality rate increased with as the volume of RBC transfusion increased. RBC transfusion volume, length of stay and ICU stay constitute independent risk factors for patient mortality during massive blood transfusion.

## Figures and Tables

**Table I:** Demographics and clinical data on various RBC transfusion volume groups.

Variable	0–4 units	5–9 units	10–14 units	15–19 units	20–24 units	25–29 units	30–39 units	≥40 units	P-value
Number of patients, n (%)	96 (6.0)	457 (28.5)	662 (41.3)	174 (10.9)	114 (7.1)	46 (2.9)	34 (2.1)	18 (1.1)	<0.001^a^
Age, years (± SD)	48.4 (23.2)	46.1 (17)	45.1 (16.4)	44.7 (17)	43 (17.8)	48.2 (16.8)	45.4 (17.8)	42.2 (16.7)	0.329^a^
Males, n (%)	43 (4.8)	210 (23.4)	390 (43.4)	109 (12.1)	79 (8.8)	31 (3.4)	27 (3)	10 (1.1)	<0.001^a^
Weight, kg (± SD)	53.9 (20)	57 (12.6)	58.4 (11.4)	58.3 (11.9)	59.2 (11.4)	59.1 (7.5)	59.9 (10.8)	56.8 (14.8)	0.158^a^
Number of patients (1)^c^, n (%)	14 (5.2)	67 (25.0)	109 (40.7)	34 (12.7)	30 (11.2)	7 (2.6)	4 (1.5)	3 (1.1)	<0.001^a^
Number of patients (2)^d^, n (%)	12 (3.1)	104 (27.2)	158 (41.3)	44 (11.5)	32 (8.4)	16 (4.2)	10 (2.6)	7 (1.8)	<0.001^a^
Number of patients (3)^e^, n (%)	68 (7.8)	267 (30.5)	358 (40.9)	91 (10.4)	47 (5.4)	21 (2.4)	17 (1.9)	7 (0.8)	<0.001^a^
Number of patients (4)^f^, n (%)	2 (2.7)	19 (25.7)	37 (50)	5 (6.8)	5 (6.8)	2 (2.7)	3 (4.1)	1 (1.4)	<0.001^a^
Clinical data (before transfusion)									
R, n/min (± SD)	20.3 (3.5)	20.2 (3.5)	20.2 (3)	20.5 (3.6)	21.9 (6)	21 (3.1)	19.9 (2.2)	19.9 (2.4)	0.012^a^
P, n/min (± SD)	90.1 (17.7)	94.9 (76)	93.1 (58.6)	87.5 (18.2)	90.7 (20.9)	93.7 (23)	118.8 (143.1)	81.1 (21.5)	0.437^a^
RP, mmHg (± SD)	112.6 (19.5)	113.6 (25.6)	113.5 (29.6)	111.3 (32.7)	109.4 (31.6)	111.3 (29.7)	114.2 (22.5)	122.5 (33.1)	0.770^a^
T,°C (± SD)	36.7 (0.7)	36.6 (1)	36.6 (0.4)	36.5 (1)	36.6 (0.6)	36.2 (1.6)	36.7 (0.5)	36.5 (0.3)	0.128^a^
RBC, ×10^12^/l (± SD)	3.6 (1.1)	3.9 (0.9)	3.9 (1.1)	3.8 (1)	3.6 (1.2)	3.7 (1.1)	4.2 (1)	4.1 (1.1)	0.023^a^
Hb, g/l (± SD)	104.6 (32.6)	116.3 (29.2)	117.1 (33.1)	120.1 (77.7)	111.8 (31)	113.9 (32.3)	127.8 (25)	128.1 (31.1)	0.059^a^
PLT, ×10^9^/l (± SD)	152.2 (88.7)	184.1 (91.7)	181.9 (102.7)	165.1 (95.1)	156.8 (86.3)	177 (99.2)	180.7 (73.8)	165.1 (104.5)	0.036^a^
PT, s (± SD)	15 (9.8)	13.8 (6.4)	13.7 (4.5)	15.5 (9.7)	13.8 (3.8)	15.5 (7.5)	13.4 (2.8)	15.9 (6.3)	0.058^a^
APTT, sec (± SD)	34.3 (11.2)	33.4 (11.8)	36.2 (26.8)	35.3 (14.3)	38.2 (27.8)	35.3 (12.4)	34.7 (6.7)	44.1 (21.7)	0.457^a^
TT, sec (± SD)	16 (4.3)	17.3 (13.8)	17.1 (5.6)	17.3 (6.6)	20.4 (13.7)	17.9 (4.8)	17.1 (2.9)	18.1 (8.1)	0.311^a^
INR (± SD)	1.4 (1)	1.4 (2.2)	1.2 (1)	1.2 (0.7)	1.4 (1.7)	1.2 (0.6)	1.1 (0.2)	1.3 (0.5)	0.957^a^
FIB, g/l (± SD)	6.4 (22.9)	12.2 (47.1)	11.7 (52.6)	6.4 (20.4)	6.3 (10.6)	21.6 (48.6)	23.1 (83.5)	2.9 (1.5)	0.574^a^
Clinical data (after transfusion)									
Days of stay (± SD)	24.5 (13.4)	25 (14.5)	28.8 (22)	29.7 (23.3)	33 (32.1)	29.8 (19)	28 (18.3)	47.6 (44.6)	<0.001^a^
Stay in ICU, days (± SD)	2.9 (3.4)	3.9 (3.5)	6.6 (10.7)	16 (53.5)	7.8 (9)	7.1 (7.3)	7.6 (12.6)	20.3 (27.5)	0.021^a^
Surgery time, h	1.4 (1.7)	2.8 (3.4)	3.5 (3.7)	3.2 (3.7)	5.2 (4.5)	3.2 (4.2)	5.7 (5.5)	4.8 (4.9)	<0.001^a^
pRBC in 24 h (units)	4	8	12	16	22	26	30	40	<0.001^b^
FFP in 24 h (units)	3	6	10	14	20	20	22	20	<0.001^b^
PLT in 24 h (units)	20	10	10	10	10	10	15	10	0.773^b^
pRBC in 72h (units)	10	8	10	16	20	22	26	26	<0.001^b^
FFP in 72h (units)	6	6	8	12	14	15	16	18	<0.001^b^
PLT in 72h (units)	16	15	10	10	20	10	10	13	0.592^b^

a Analysis used variance;

b values are medians, analysis used Kruskall-Wallis test;

c patients who suffered from trauma;

d patients with cardiopathy;

e patients who underwent general surgery;

f obstetric patients.

R, respiration; P, pulse; RP, resting pressure; T, temperature; RBC, red blood cell; HB, hemoglobin; PLT, platelets; PT, prothrombin time; APTT, activated partial thromboplastin time; TT, thrombin time; INR, international normalized ratio; FIB, fibrinogen; ICU, intensive care unit; pRBC, packed red blood cells; FFP, fresh frozen plasma; PLT, platelets; SD, standard deviation.

**Table II:** Mortality and survival rates in various RBC transfusion volume groups [n (%)].

Duration	Outcome	0–4 units	5–9 units	10–14 units	15–19 units	20–24 units	25–29 units	30–39 units	≥40 units	Total	P-value
24 h	Mortality	7 (7.3)	17 (3.7)	40 (6.0)	19 (10.9)	20 (17.5)	14 (30.4)	8 (23.5)	7 (38.9)	132 (8.2)	0.001
	Survival	89 (92.7)	440 (96.3)	622 (94.0)	155 (89.1)	94 (82.5)	32 (69.6)	26 (76.5)	11 (61.1)	1469 (91.8)	
72 h	Mortality	21 (9.7)	7 (2.3)	29 (5.2)	20 (9.1)	16 (13.4)	11 (15.5)	14 (21.5)	14 (28.0)	132 (8.2)	0.001
	Survival	195 (90.3)	299 (97.7)	525 (94.8)	200 (90.9)	103 (86.6)	60 (84.5)	51 (78.5)	36 (72.0)	1469 (91.8)	

RBC, red blood cell.

**Table III:** Results of multivariate logistic regression analysis.

Variable	β	SE	P-value	Odds ratio	95.0% CI
RBC volume	−0.65	0.10	<0.001	0.52	0.43–0.64
Length of stay	1.02	0.38	0.01	2.79	1.31–5.92
ICU stay	−0.84	0.36	0.02	0.43	0.21–0.88
Constant	2.14	1.00	0.03	8.48	

SE, standard error; CI, confidence interval; RBC, red blood cell; ICU, intensive care unit.
